# Domain swap facilitates structural transitions of spider silk protein C‐terminal domains

**DOI:** 10.1002/pro.4783

**Published:** 2023-10-16

**Authors:** Charlotte Rat, Cedric Heindl, Hannes Neuweiler

**Affiliations:** ^1^ Department of Biotechnology & Biophysics Julius‐Maximilians‐University Würzburg Würzburg Germany

**Keywords:** domain swap, folding intermediates, protein folding, spider silk

## Abstract

Domain swap is a mechanism of protein dimerization where the two interacting domains exchange parts of their structure. Web spiders make use of the process in the connection of C‐terminal domains (CTDs) of spidroins, the soluble protein building blocks that form tough silk fibers. Besides providing connectivity and solubility, spidroin CTDs are responsible for inducing structural transitions during passage through an acidified assembly zone within spinning ducts. The underlying molecular mechanisms are elusive. Here, we studied the folding of five homologous spidroin CTDs from different spider species or glands. Four of these are domain‐swapped dimers formed by five‐helix bundles from spidroins of major and minor ampullate glands. The fifth is a dimer that lacks domain swap, formed by four‐helix bundles from a spidroin of a flagelliform gland. Spidroins from this gland do not undergo structural transitions whereas the others do. We found a three‐state mechanism of folding and dimerization that was conserved across homologues. In chemical denaturation experiments the native CTD dimer unfolded to a dimeric, partially structured intermediate, followed by full unfolding to denatured monomers. The energetics of the individual folding steps varied between homologues. Contrary to the common belief that domain swap stabilizes protein assemblies, the non‐swapped homologue was most stable and folded four orders of magnitude faster than a swapped variant. Domain swap of spidroin CTDs induces an entropic penalty to the folding of peripheral helices, thus unfastening them for acid‐induced unfolding within a spinning duct, which primes them for refolding into alternative structures during silk formation.

## 1 INTRODUCTION

Silk fibers from orb‐web weaving spiders belong to the toughest materials in nature (Gosline et al., 1999; Heim et al., 2009; Vollrath and Knight, 2001). Web spiders use up to seven specialized glands to spin silk threads for various tasks including prey capture, reproduction, and shelter (Rising and Johansson, 2015; Vollrath and Knight, 2001). Spider silk is a protein‐based biopolymer that consists of fibroins, so‐called spidroins. Despite the differences in mechanical properties of the different silks they produce, the process of synthesis by the animal is conserved. At the beginning of the process, spidroins are stored as soluble proteins at high concentration in the ampulla of a gland where they form a viscous spinning dope (Heim et al., 2009; Vollrath and Knight, 2001). On demand, the dope passes through a narrowing, S‐shaped duct where spidroins experience a series of mechanical and chemical stimuli (Heim et al., 2009; Rising and Johansson, 2015; Vollrath and Knight, 2001). Shear forces align spidroins (Vollrath and Knight, 2001) and changes in salt composition and pH induce phase and structural transitions (Heim et al., 2009; Rising and Johansson, 2015). At the end of the duct, the solid silk fiber emerges from the exit spigot.

Raman spectromicroscopy reveals gland‐specific differences in structural transitions of spidroins during their transformation into silk (Lefevre et al., 2011). Spidroins from major (Ma) and minor (Mi) ampullate glands, which form dragline and auxiliary silk, are natively unfolded under storage conditions. In the corresponding silks they are transformed into highly oriented β‐sheet or α/β‐structures. By contrast, spidroins from the flagelliform (Flag) gland, which forms capture spiral silk, undergo no structural transitions: they are highly disordered both under storage conditions and in solid silk (Lefevre et al., 2011). These remarkable differences in structural transformations are thought to originate from differences in sequence (Challis et al., 2006; Lefevre et al., 2011). The molecular details are only understood in parts. They are of interest for material scientists trying to infer mechanical properties from protein structural architectures, interactions, and energetics (Yarger et al., 2018). They are also of interest for biochemists trying to understand fundamental mechanisms of protein folding and protein structure–function relationships.

The amino acid composition of spidroins is unusual compared to the one of conventional proteins (Vollrath and Knight 2001). The bulk of a spidroin consists of an intrinsically disordered central domain that contains repetitive peptide motifs of simple amino acid composition, typically enriched in Ala, Gly, Gln, Pro, and Ser (Heim et al., 2009; Vollrath and Knight, 2001). The unusual amino acid composition of the central domain extends into the globular folded N‐ and C‐terminal domains (NTD and CTD). NTD and CTD are five‐helix bundles that provide water‐solubility under storage conditions and connectivity in silk (Askarieh et al., 2010; Hagn et al., 2010). Sequences of the spidroin terminal domains are highly conserved indicating conserved and important functional roles (Eisoldt et al., 2012). Both NTD and CTD form all‐helical homo‐dimers, respectively, but their quaternary structures are very different (Askarieh et al., 2010; Hagn et al., 2010). They contain structural switches that are triggered by chemical stimuli within spinning ducts. One of these stimuli is a drop of solution pH from 7.6 in the ampulla to 5.7, and possibly below, at the end of the duct, which affects NTD and CTD differently (Andersson et al., 2014). The NTD is a monomer under storage conditions and forms a tight dimer upon solution acidification, a process that involves electrostatic interactions and conformational change (Askarieh et al., 2010; Gaines et al., 2010; Hagn et al., 2011; Heiby et al., 2019; Jaudzems et al., 2012; Kronqvist et al., 2014; Ries et al., 2014; Schwarze et al., 2013). NTD dimerization is thought to polymerize spidroins. The CTD, on the other hand, is a dimer already under storage conditions connecting two spidroins permanantly (Hagn et al., 2010). During passage of spidroins through an acidified zone within the spinning duct the CTD gets destabilized (Andersson et al., 2014). Subsequent partial unfolding of the CTD induces formation of β‐sheets, which is the dominant secondary structure in spider silk. The process is associated with phase transition (Andersson et al., 2014; Bauer and Scheibel, 2017; Gauthier et al., 2014; Hagn et al., 2010).

The sequences of CTDs are less conserved compared to the ones of NTDs (Eisoldt et al., 2012). However, a pH sensitive module is conserved across the entire spider silk gene family (Strickland et al., 2018). Structures of CTDs from Ma, Mi, and aciniform glands are highly similar and exhibit a characteristic domain swap where the C‐terminal helices within the dimer exchange between monomer subunits thus forming a macromolecular clamp (Andersson et al., 2014; Gao et al., 2013; Hagn et al., 2010; Wang et al., 2014). Within the family of CTDs, sequences from the Flag gland differ (Challis et al., 2006). A recently determined structure of a Flag spidroin CTD lacks domain swap but its secondary and tertiary structure is otherwise similar (Li et al., 2022).

Domain swap is a phenomenon in protein dimerization where monomers exchange parts of their structure upon association. It was first discovered by Eisenberg and colleagues and suggested as an evolutionary mechanism in protein oligomerization (Anonymous, 1994; Bennett et al., 1994). Domain swap increases the area of a dimerization interface considerably and is thus suggested to exert a marked stabilizing effect on protein assemblies (Mackinnon et al., 2013; Newcomer, 2002). It is further thought to regulate protein function and aggregation (Newcomer, 2002; Rousseau et al., 2012), and plays an important role in β‐amyloid fibril formation (Nelson and Eisenberg, 2006). The folding mechanism of domain swap and how it serves to regulate function are elusive (Gronenborn, 2009).

Folding of spidroin CTDs from various glands and species has been studied and is reported to proceed via a two‐state transition, but with indications for or the observation of residual structure in denatured states (Andersson et al., 2014; Bauer and Scheibel, 2017; Gao et al., 2013; Gauthier et al., 2014; Hagn et al., 2010). In these studies, urea was applied as denaturant in chemical denaturation experiments. The application of urea led to apparently incomplete unfolding, hampering mechanistic, quantitative studies. An exception was a spidroin CTD from the Ma gland of the nursery web spider *Euprosthenops australis* that fully unfolded in urea and was found to self‐assemble via a three‐state mechanism (Rat et al., 2018).

Here, we investigated the folding and dimerization of a family of five homologous spidroin CTDs originating from various species or glands in a comparative manner. We applied the stronger chaotrope guanidinium chloride (GdmCl) instead of urea. We observed full unfolding of all CTDs, which allowed us to explore their entire free energy surfaces. We found a three‐state mechanism of folding and dimerization via a dimeric folding intermediate that was conserved across homologues. The conformational stabilities of native and intermediate states varied between homologues. The CTD from the Flag gland that lacked domain swap was most stable and showed fast kinetics of folding. The observed differences can be explained by the entropic penalty associated with domain swap with functional consequences.

## 2 RESULTS

### 2.1 Compilation of a family of spidroin CTDs

**FIGURE 1:**
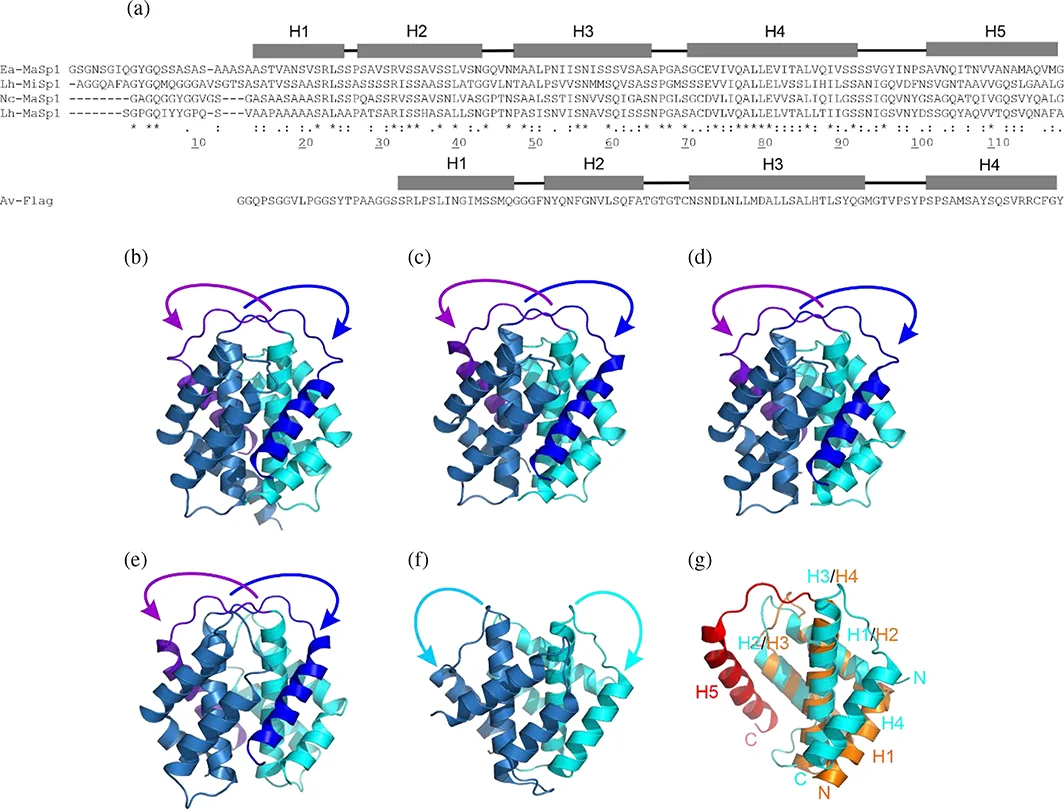
Structures of homologous spidroin CTDs. (a) Sequence alignment of Ea‐MaSp1‐CTD, Nc‐MaSp1‐CTD, Lh‐MaSp1‐CTD, and Lh‐MiSp1‐CTD (Align tool, UniProt resource). Identical (*), very similar (:), and similar (.) residues are indicated at the bottom. The sequence numbering scheme refers to Lh‐MaSp1‐CTD. Segments forming helices 1–5 (H1–H5) are indicated at the top. The sequence of Av‐Flag‐CTD is shown at the bottom with helices H1–H4 indicated. (b–d) Structural homology models of Ea‐MaSp1‐CTD (b), Nc‐MaSp1‐CTD (c), and Lh‐MaSp1‐CTD (d), built using the structure of *A. diadematus* as a template (PDB ID: 2KHM, SWISS‐MODEL server, University of Basel, Switzerland). (e) Structural homology model of Lh‐MiSp1‐CTD built using the structure of the Mi‐Sp1‐CTD from *T. antipodiana* as a template (PDB ID: 2M0M). (f) NMR solution structure of the Av‐Flag‐CTD (PDB ID: 7VU7). Monomer subunits are colored light blue and cyan. C‐terminal helices that swap domains in Ma and Mi spidroin CTDs are highlighted dark blue and purple. The swap is indicated by arrows. The corresponding helices remaining on their subunits in the Av‐Flag‐CTD are indicated by arrows. (g) Structural alignment of the monomer subunit of the Av‐Flag‐CTD (cyan) with the one of a MiSp1‐CTD (orange) (PDB IDs 7VU7 and 2M0M). Helices H1–H4 and H1–H5, respectively, are indicated. N‐ and C‐terminal ends are indicated (N and C). Helix H5 that swaps domains in MiSp1‐CTD is highlighted red.

The selection of CTDs was guided by our previous study of a family of homologous NTDs (Heiby et al., 2017), with the aim to obtain complementary results from the opposite terminal ends of same spidroins. We selected the CTDs from Ma glands of the black widow spider *Latrodectus hesperus* (Lh), the golden silk orb‐weaver *Nephila clavipes* (Nc), and the nursery web spider *Euprosthenops australis* (Ea). We further chose to investigate the homologue from the minor ampullate (Mi) gland of Lh to compare two CTDs from the same species but different glands. We finally included the recently reported CTD from the Flag gland of the large nocturnal spider *Araneus ventricosus* (Av) (Li et al., 2022). We named the five CTDs Lh‐MaSp1‐CTD, Nc‐MaSp1‐CTD, Ea‐MaSp1‐CTD, Lh‐MiSp1‐CTD, and Av‐Flag‐CTD, respectively. The sequences of Lh‐MaSp1‐CTD, Lh‐MiSp1‐CTD, Nc‐MaSp1‐CTD, and Ea‐MaSp1‐CTD were highly similar with a mean identity of 48 ± 7% (Align tool, UniProt resource, Figure 1a). The Av‐Flag‐CTD had a lower sequence identity of 23 ± 3% compared to the others and a shorter N‐terminus lacking helix 1 (Li et al., 2022). Indeed, sequence analysis of silk genes indicates that helix 1 is absent in some CTDs (Strickland et al., 2018) including the Av‐Flag‐CTD (Li et al., 2022). Ma CTDs and the Flag CTD contain a conserved Cys residue on the central helix of the dimerization interface, which forms a covalent disulfide linkage between the two monomer subunits. This Cys residue is missing in CTDs from Mi glands (Li et al., 2022) and a disulfide bond is not required for silk formation (Ittah et al., 2007).

We built structural homology models of Ea‐MaSp1‐CTD, Nc‐MaSp1‐CTD, Lh‐MaSp1‐CTD, and Lh‐MiSp1‐CTD. Models were built using the SWISS‐MODEL server (University of Basel, Switzerland). The sequences of Ea‐MaSp1‐CTD, Nc‐MaSp1‐CTD, and Lh‐MaSp1‐CTD show high identities of 59%, 76%, and 56%, respectively, compared to the sequence from *A. diadematus* for which an experimental structure is available (PDB ID: 2KHM) (Hagn et al., 2010). The sequence of Lh‐MiSp1‐CTD showed 66% identity compared to the one from *T. antipodiana* (PDB ID: 2M0M) (Gao et al., 2013). These sequence identities are far above the 30%‐threshold criterion for successful homology modeling (Chothia and Lesk, 1986; Xiang, 2006) and higher than the 50%‐threshold for high‐accuracy structure prediction with root mean square errors for main‐chain atoms of about 1 Å (Baker and Sali, 2001). We were thus able to build accurate homology models (Figure 1b–e). The structure of the Av‐Flag‐CTD was determined recently using solution NMR spectroscopy (Li et al., 2022) (Figure 1f). Comparison of structures of the five CTDs shows that the most striking difference is the lack of domain swap in Av‐Flag‐CTD (Li et al., 2022). Otherwise, the tertiary structure is conserved showing superposition of helices (Figure 1g).

### 2.2 Conservation of three‐state folding and dimerization with varying energetics

We synthesized spidroin CTDs through heterologous overexpression in *E. coli* bacterial cells followed by their isolation using chromatographic methods. In the synthesis of the Av‐Flag‐CTD we found that formation of the inter‐molecular covalent dimer, that is, formation of the disulfide between monomers, was extremely slow, which contrasted with the other CTDs. The observation was in agreement with previous reports where formation of the covalent dimer of the Av‐Flag‐CTD required more than 1 week (Li et al., 2022). In this homologue, the presence of an additional, non‐conserved Cys residue at the C‐terminus leads to formation of oligomers. These can be eliminated through mutation C105S that replaces the C‐terminal thiol by a hydroxy group and retains structure and function of the homologue (Li et al., 2022). Indeed, we found that synthesis of Av‐Flag‐CTD mutant C105S yielded a pure and homogeneous CTD dimer. This contrasted with the wild type that showed incomplete covalent dimerization and the presence of oligomers besides dimers, in agreement with previous results (Li et al., 2022) (Figure S1). We proceeded to use mutant C105S as a pseudo wild‐type Av‐Flag‐CTD throughout our experiments.

We recorded far‐UV circular dichroism (CD) spectra of all five homologues in pH 7.0 buffered solutions, conditions similar to the storage conditions of spidroins in ampullas of spinning glands (Andersson et al., 2014). The spectra were characteristic of properly folded, all‐helical domains (Figure S2). The smaller CD signal amplitude of the Av‐Flag‐CTD compared to its homologues is explained by the lack of N‐terminal helix 1.

**FIGURE 2:**
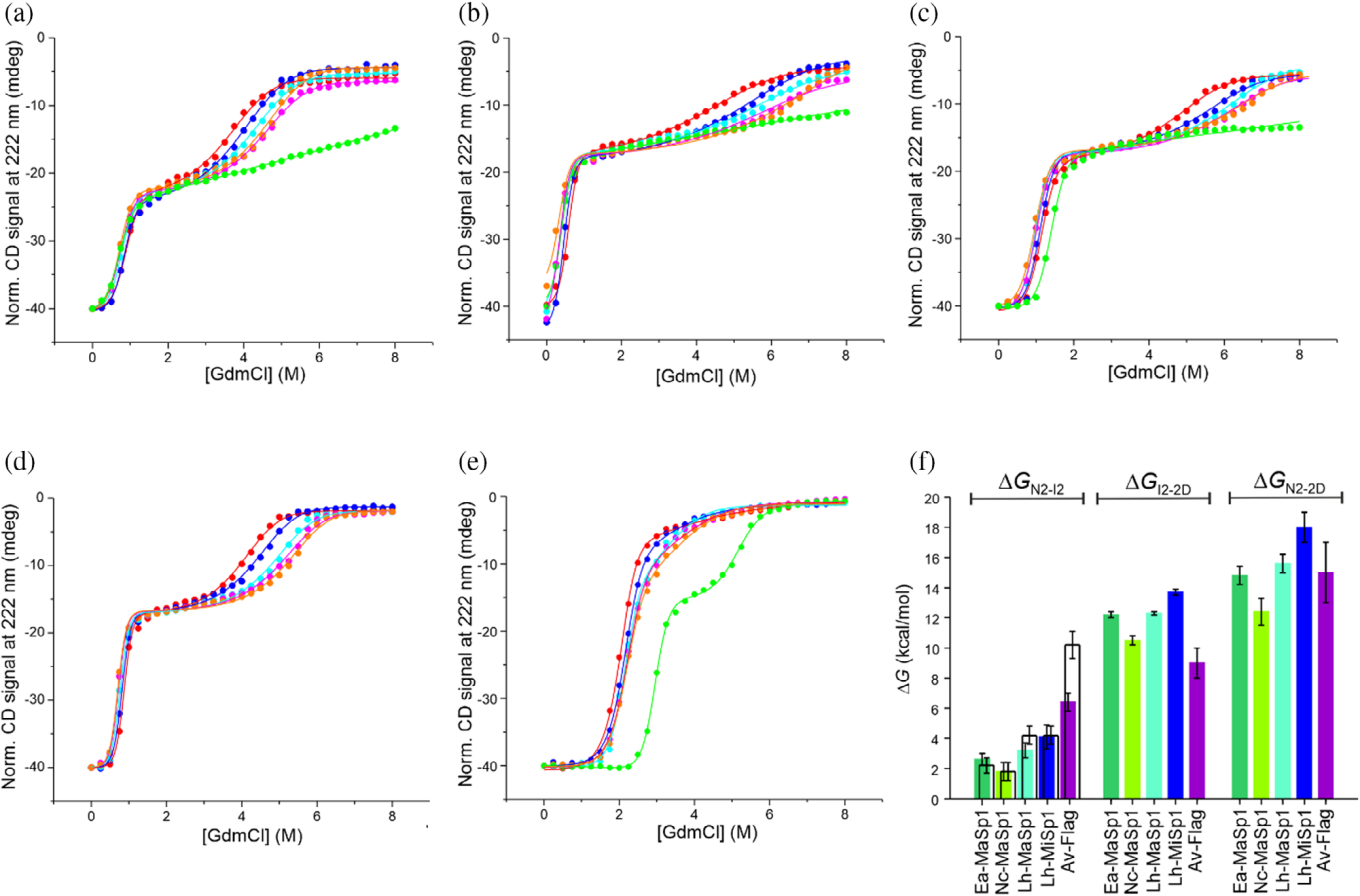
Chemical equilibrium denaturation of spidroin CTDs. Equilibrium denaturation data of Ea‐MaSp1‐CTD (a), Nc‐MaSp1‐CTD (b), Lh‐MaSp1‐CTD (c), Lh‐MiSp1‐CTD (d), and Av‐Flag‐CTD (e) measured using GdmCl as denaturant, at 25°C in pH 7.0 buffered solutions. Data measured under oxidizing conditions from MaSp1‐CTDs and the Av‐Flag‐CTD are shown in green. Data measured under reducing conditions and at varying protein concentrations are shown in red (5 μM CTD), blue (10 μM CTD), cyan (20 μM CTD), magenta (30 μM CTD), and orange (40 μM CTD). An exception was the Lh‐MiSp1 CTD that was measured under oxidizing conditions at varying protein concentrations (same color code applies). Data were normalized to the native‐state signal amplitude of −40 mdeg for reasons of clarity. Solid lines are fits to the data using a thermodynamic model for a mono‐molecular two‐state transition, N_2_ ↔ I_2_, that applied to Ea‐MaSp1‐CTD, Nc‐MaSp1‐CTD, and Lh‐MaSp1‐CTD under oxidizing conditions, a mono‐molecular three‐state transition, N_2_ ↔ I_2_ ↔ D_2_, that applied to the Av‐Flag‐CTD under oxidizing conditions, and a bimolecular three‐state transition, N_2_ ↔ I_2_ ↔ 2D, that applied to Ea‐MaSp1‐CTD, Nc‐MaSp1‐CTD, Lh‐MaSp1‐CTD, and Lh‐MaSp1‐CTD under reducing conditions, as well as to the Lh‐MiSp1‐CTD measured under oxidizing conditions. (f) Free energies calculated from fits to the data. Values determined from data recorded in oxidizing solutions are shown as black, open bars. Values determined from denaturation data recorded in reducing solutions are shown as colored, closed bars, except for the Lh‐MiSp1‐CTD that was measured under oxidizing conditions.

We performed equilibrium chemical denaturation experiments using far‐UV CD spectroscopy probing the loss of α‐helical secondary structure. Experiments were performed under both oxidizing and reducing solution conditions, that is, in the absence and presence of dithioerythritol (DTE). Under reducing solution conditions disulfide bonds were cleaved yielding non‐covalent CTD dimers. Under oxidizing conditions, the monomer subunits were covalently linked through disulfide bonds. The Lh‐MiSp1‐CTD lacked native Cys and was thus studied under oxidizing solution conditions only. We applied guanidinium chloride (GdmCl) as a strong denaturant to achieve full unfolding of CTDs.

Under oxidizing conditions, as in the animals spinning glands, Ea‐MaSp1‐CTD, Nc‐MaSp1‐CTD, and Lh‐MaSp1‐CTD showed two‐state unfolding transitions to structured denatured states (Figure 2a–c). The amount of residual structure was ~40%–50% of native states as judged by the relative far‐UV CD signal amplitudes. The Nc‐MaSp1‐CTD was least stable showing hardly a native‐state baseline (Figure 2b). The positive slope of the denatured‐state baseline of Ea‐MaSp1‐CTD indicated further unfolding of this homologue (Figure 2a). The Lh‐MiSp1‐CTD showed a three‐state unfolding transition with clearly separable native, intermediate, and denatured states under oxidizing conditions (Figure 2d). It appeared that the lack of native Cys and thus the lack of a covalent disulfide bond between monomers reduced the stability of this homologue such that full unfolding became possible. The disulfide in Ma homologues, on the other hand, stabilized them to an extent that they never fully unfolded. Interestingly, the Av‐Flag‐CTD showed full three‐state unfolding despite the presence of an intermolecular disulfide (Figure 2e). We concluded that disulfides of Ma homologues, but not of the Av‐Flag‐CTD, were responsible for preserving a structured denatured state even at high concentrations of GdmCl. To obtain energetics of the first unfolding transitions, denaturation data of MaSp1 CTDs were fitted using a thermodynamic model for a mono‐molecular two‐state transition, N_2_ ↔ I_2_, where N_2_ was the native covalent dimer and I_2_ was the covalent dimeric intermediate. The data of the Lh‐MiSp1‐CTD were fitted using a three‐state thermodynamic model for dimer unfolding, N_2_ ↔ I_2_ ↔ 2D, where N_2_ and I_2_ were the dimeric native and intermediate states and D was the denatured monomer. The data of the Av‐Flag‐CTD were fitted using a mono‐molecular model of three‐state folding, N_2_ ↔ I_2_ ↔ D_2_, where N_2_, I_2_, and D_2_ were dimeric native, dimeric intermediate, and dimeric denatured states, respectively. Details of thermodynamic modeling are provided in the Section 4. The thermodynamic parameters of unfolding obtained from denaturation measured under oxidizing conditions are listed in Table S1. The change of free energies associated with unfolding are collated in Figure 2f. The native state of the Av‐Flag‐CTD was more than twice as stable than the ones of the other homologues.

We next performed denaturation experiments under reducing solution conditions using GdmCl as denaturant to achieve full unfolding of all homologues. Under these conditions the homologues showed three‐state unfolding transitions to fully denatured states (Figure 2a–e). Although the shape of the denaturation curve of the Av‐Flag‐CTD appeared two‐state, a thermodynamic model for dimer two‐state unfolding (Hobart et al. 2002), N_2_ ↔ 2D, did not describe the data accurately (Figure S3a). Instead, the data were well described by a three‐state model containing a dimeric intermediate (Hobart et al., 2002), N_2_ ↔ I_2_ ↔ 2D, that also applied to the other homologues (Figure S3b, Figure 2a–e). To investigate the nature of the intermediate further, that is, to assess whether it was a dimer or a monomer, we varied the protein concentration in chemical denaturation experiments. Since dimerization is a bimolecular event it obeys the law of mass action. Thus, the energetics of folding associated with the dimerization event will depend on protein concentration (Neet and Timm, 1994). Protein concentration‐dependent denaturation curves were measured under reducing solution conditions facilitating formation and dissociation of dimers during equilibrium denaturation. Lh‐MiSp1‐CTD was measured under oxidizing conditions because it lacked a native disulfide. All denaturation data showed a shift of the second transition to higher denaturant mid‐point concentrations ([GdmCl]_50%_) with increasing concentration of protein (Figure 2a–e). The first transitions remained invariant instead. The results thus identified the first transitions as mono‐molecular folding/unfolding events and the second to involve the dimerization step. We could thus assign with confidence a three‐state mechanism of folding and dimerization via a dimeric intermediate, N_2_ ↔ I_2_ ↔ 2D. The ellipticities of the structured denatured states observed in two‐state denaturation measured under oxidizing conditions matched the ones of the intermediate states in three‐state folding measured under reducing conditions, indicating that both states reflected the same molecular species (Figure 2a–e). Three‐state unfolding was less obvious in data recorded from the Av‐Flag‐CTD because first and second transitions appeared to merge at low concentrations of protein. Thermodynamic parameters of folding and dimerization obtained from fits to the data using the three‐state model, N_2_ ↔ I_2_ ↔ 2D, are summarized in Table S2. The changes of unfolding free energies measured under reducing conditions are collated in Figure 2f. The native states of Ea‐MaSp1‐CTD, Nc‐MaSp1‐CTD, Lh‐MaSp1‐CTD, and Lh‐MiSp1‐CTD were more labile than the one of the Av‐Flag‐CTD, similar as observed under oxidizing conditions. Under reducing solution conditions, the free energies of folding, Δ*G*_N2‐I2_, ranged between 2 and 4 kcal mol^−1^ for Ea‐MaSp1‐CTD, Nc‐MaSp1‐CTD, Lh‐MaSp1‐CTD, and Lh‐MiSp1‐CTD, respectively. The higher value of Δ*G*_N2‐I2_ of the Av‐Flag‐CTD was compensated by a reduced stability of the intermediate, that is, a smaller value of Δ*G*_I2‐2D_. As a result, the total free energy of folding and dimerization (Δ*G*_N2‐D_ = Δ*G*_N2‐I2_ + Δ*G*_I2‐D_) was largely conserved across the homologues (Figure 2f). The lack of a native‐state baseline in denaturation data of the Nc‐MaSp1‐CTD indicated that this homologue was most unstable and barely folded even in solutions without denaturant. Interestingly, the NTD of the same spidroin was also least stable within the family of homologous spidroin NTDs (Heiby et al., 2017). The result supports the view that sequence properties from the central domains, which are the bulk of a spidroin, extend into both terminal ends, that is, properties of the terminal domains evolved from the central segments. It is important to note that the stabilities of spidroin terminal domains inferred from denaturation experiments in vitro may further be modulated by a variation of spider and gland‐specific solution conditions in vivo.

### 2.3 CTDs respond differently to solution acidification

**FIGURE 3:**
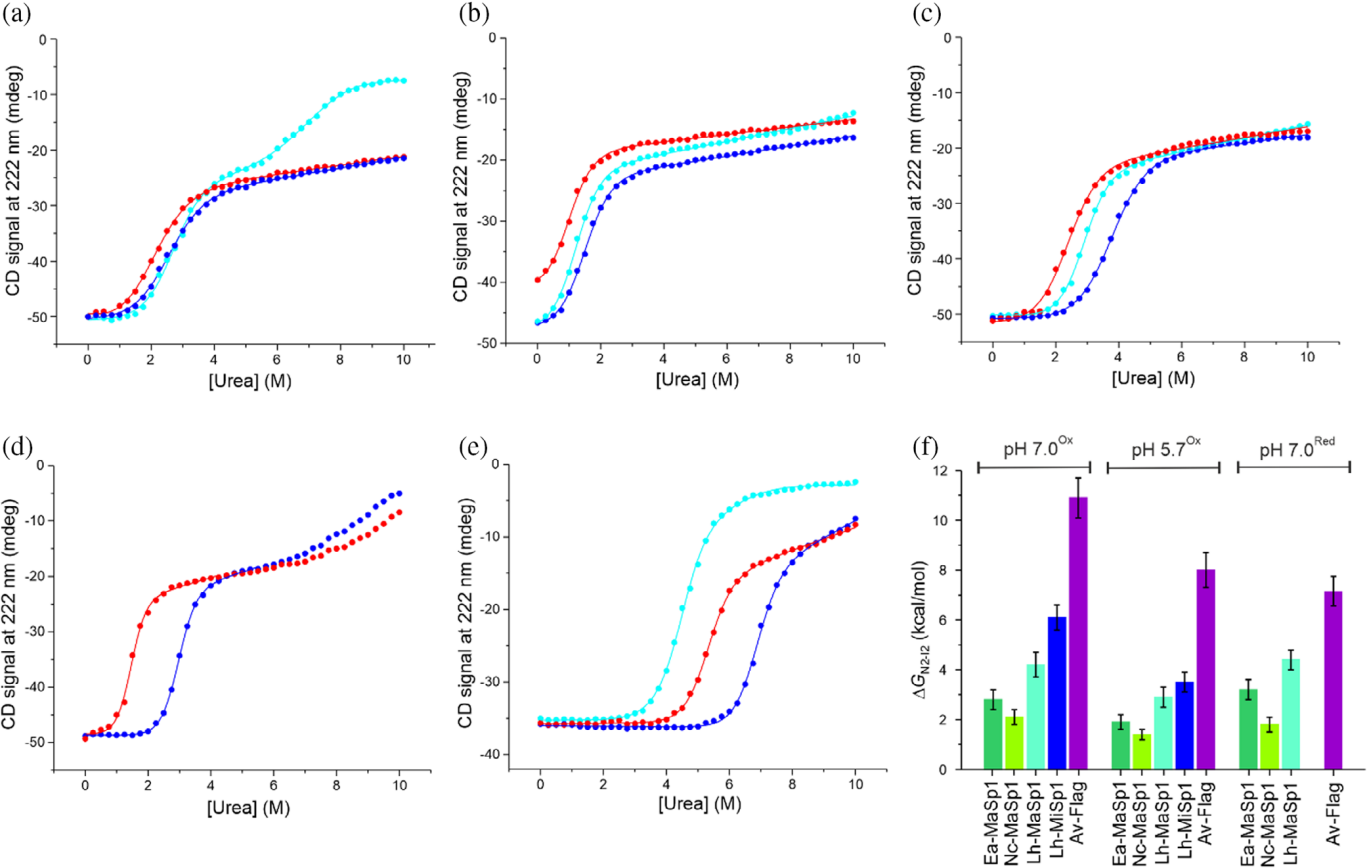
Chemical equilibrium denaturation of CTDs measured at pH 7.0 and pH 5.7 using urea as denaturant. Equilibrium denaturation of 20 μM Ea‐MaSp1‐CTD (a), Nc‐MaSp1‐CTD (b), Lh‐MaSp1‐CTD (c), Lh‐MiSp1‐CTD (d), and Av‐Flag‐CTD (e). Data recorded under oxidizing conditions at pH 7.0 and at pH 5.7 are shown in blue and in red, respectively. Data recorded under reducing conditions at pH 7.0 are shown in cyan. Solid lines are fits to the data using the thermodynamic model for a two‐state transition, N_2_ ↔ I_2_, except of Ea‐MaSp1‐CTD and Av‐Flag‐CTD measured under reducing conditions where data were described by a three‐state model, N_2_ ↔ I_2_ ↔ 2D. (f) Free energies of unfolding (Δ*G*_N2‐I2_) under oxidizing conditions at pH 7.0 (pH 7.0^Ox^), under oxidizing conditions at pH 5.7 (pH 5.7^Ox^), and under reducing conditions at pH 7.0 (pH 7.0^Red^), determined from fits to the data.

A drop of solution pH from 7.0 to 5.7 along the spinning duct destabilizes CTDs (Andersson et al., 2014). Their partial unfolding is thought to induce structural transitions in spidroins during silk formation (Andersson et al., 2014; Bauer and Scheibel, 2017; Eisoldt et al., 2012; Gauthier et al., 2014; Hagn et al., 2010). We investigated the effect of the change of pH on folding and native‐state stabilities of the five homologous CTDs. We performed chemical denaturation experiments under oxidizing conditions both at pH 7.0 and at pH 5.7, resembling the conditions in the ampulla of the gland and at the end of the duct (Andersson et al., 2014). In denaturation experiments above, using GdmCl as denaturant, we found overall low stabilities of native states of Ma and Mi spidroin CTDs and an early onset of unfolding (Figure 2a–e). We therefore applied the milder denaturant urea in denaturation experiments where we destabilized the CTDs through a drop of pH to extend their native‐state baselines. We observed unfolding to structured denatured states in urea, similar as observed in GdmCl titrations, both at pH 7.0 and at pH 5.7 (Figure 3a–e). The unfolding to structured denatured or intermediate states was well described by a mono‐molecular two‐state model. The Lh‐MiSp1‐CTD and the Av‐Flag‐CTD unfolded further at high concentrations of urea without reaching a denatured‐state baseline (Figure 3d,e). The thermodynamic parameters of unfolding are summarized in Table S3. The free energies of unfolding are collated in Figure 3f. Conformational stabilities of MaSp1 CTDs at pH 7.0 obtained from urea titrations compared well with the ones obtained from GdmCl titrations. The value of Δ*G*_N2‐I2_ of Lh‐MiSp1‐CTD obtained from urea titrations was higher compared to the value obtained from GdmCl titrations, which may be explained by a better defined native‐state baseline in the urea titration (Figures 2d and 3d). The conformational stability of the Av‐Flag‐CTD exceeded the ones of the other homologues by a large margin (Figure 3f, Table S3). The drop of pH from 7.0 to 5.7 reduced native‐state stabilities by about 30%–40% (Figure 3f, Table S3). The result is in qualitative agreement with previously reported thermal denaturation data of the Lh homologue (Bauer and Scheibel, 2017). The Nc‐MaSp1‐CTD lost some of its structure upon the drop of pH from 7.0 to 5.7. This was evident from a reduced native‐state ellipticity observed at pH 5.7 compared to at pH 7.0 (Figure 3b). The finding is in agreement with a molten globule at low pH reported for this homologue (Gauthier et al., 2014).

We next investigated the contribution of the intermolecular disulfide to conformational stabilities. To this end, we recorded denaturation data at pH 7.0 under reducing conditions using urea as denaturant and compared results to the ones obtained under oxidizing conditions (Figure 3). Under reducing conditions the Ea‐MaSp1‐CTD showed three‐state unfolding, as reported previously (Rat et al., 2018) (Figure 3a). The Av‐Flag‐CTD showed unfolding to a virtually fully denatured state containing very little residual ellipticity. Data were well described by three‐state model of dimer unfolding, N_2_ ↔ I_2_ ↔ 2D (Figure 3e), as we found in GdmCl titrations above. Lh‐MaSp1‐CTD and Nc‐MaSp1‐CTD showed two‐state transitions to structured denatured states that resembled the intermediate states detected in GdmCl titrations, that is, removal of the disulfide bond did not destabilize these CTDs sufficiently to achieve full unfolding. Our analysis showed that native‐state stabilities of Ea‐MaSp1‐CTD, Lh‐MaSp1‐CTD, and Nc‐MaSp1‐CTD were not significantly influenced by formation of the disulfide, that is, values of Δ*G*_N2‐I2_ measured under oxidizing and reducing conditions at pH 7.0 were within error (Figure 3f). For Lh‐MaSp1‐CTD, the decrease of the transition midpoint ([urea]_50%_) was compensated by an increase of equilibrium *m*‐value (*m*_N2‐I2_), yielding similar stabilities (Table S3). Removal of the disulfide bond in the Av‐Flag‐CTD destabilized this homologue by ~35% (Figure 3f, Table S3).

### 2.4 Kinetics of folding of the Av‐Flag‐CTD

**FIGURE 4:**
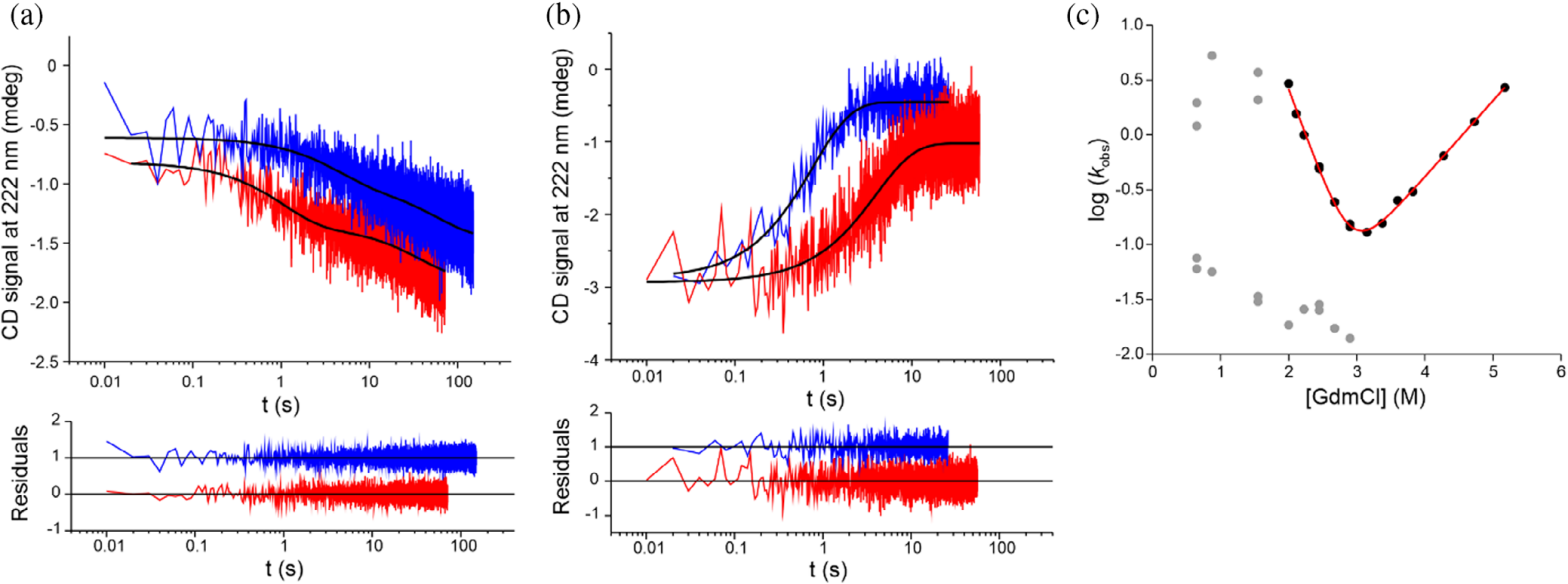
Kinetics of folding of the Av‐Flag‐CTD. (a) Representative kinetic transients of folding measured in 2.4 M GdmCl (red) and in 2.9 M GdmCl (blue). Black lines are bi‐exponential fits to the data. (b) Representative kinetic transients of unfolding measured in 4.0 M GdmCl (red) and in 5.25 M GdmCl (blue). Measurements were done at pH 7.0 under oxidizing conditions. Black lines are mono‐exponential fits to the data. Residual plots are below each graph in same color code. Residuals shown in blue are offset by +1 for reasons of clarity. (c) Observed rate constants (*k*_obs_) plotted versus denaturant concentration. The red line is a fit to the black data using the kinetic model for a barrier‐limited two‐state transition (chevron analysis). Data points shown in gray are the observed rate constants from the second exponential decays in the transients of refolding and of the rollover in the folding arm of the chevron plot at concentrations below 2 M GdmCl.

To gain further insights into origins of the high stability of the Av‐Flag‐CTD we measured its kinetics of folding using stopped‐flow far‐UV CD spectroscopy. Kinetic transients of folding/unfolding were measured by rapidly mixing native or chemically denatured Av‐Flag‐CTD samples into pH 7.0 buffered solutions containing various concentrations of GdmCl. To avoid complications in data analysis arising from a monomer/dimer equilibrium, measurements were carried out under oxidizing conditions where the CTD was a covalent dimer. We used GdmCl as denaturant instead of urea to avoid the application of very high concentrations of denaturant that lead to an increase of solution viscosity and can cause precipitation of denaturant in the stopped‐flow apparatus. In kinetic transients of folding measured at concentrations of up to 3 M GdmCl we observed bi‐exponential decays of the CD signal (Figure 4a). A mono‐exponential fit was not sufficient to describe the data accurately (Figure S4). The rate constant of the second, slow exponential phase reduced progressively at higher concentrations of denaturant and entered the minutes time scale at >3 M GdmCl. Kinetic transients of unfolding measured at concentrations >3 M were well described by mono‐exponential functions (Figure 4b). The origin of the slow kinetic phase may be cis/trans isomerization of proline, which occurs on a seconds to minutes time scale (Brandts et al., 1975): the sequence of the Av‐Flag‐CTD contains seven proline residues (Figure 1a). Disappearance of the slow exponential phase at high concentrations of denaturant may be explained by the fact that it moved beyond the upper time limit of our stopped‐flow setup that was ~100 s. The observed rate constants of the fast kinetic phase varied with denaturant concentration following the shape of a chevron, which is characteristic of a barrier‐limited two‐state transition of folding (Jackson and Fersht, 1991) (Figure 4c). There was a rollover of data in the folding arm at concentration below 2 M GdmCl. This could originate from a populated folding intermediate, the presence of proline residues (Brandts et al. 1975), or reflect the limited time resolution of our stopped‐flow setup, which suffered from the inherently low signal‐to‐noise of a far‐UV CD signal. All kinetic parameters inferred from fits to the data are provided in Table S4.

Chevron analysis revealed a microscopic rate constant of folding and unfolding of *k*_f_ = 6400 ± 1900 s^−1^ and *k*_u_ = 7 ± 1 × 10^−4^ s^−1^. The microscopic *m*‐values of folding and unfolding were *m*_f_ = 2.3 ± 0.1 kcal mol^−1^ M^−1^ and *m*_u_ = 1.0 ± 0.1 kcal mol^−1^ M^−1^. The free energy calculated from kinetics was Δ*G* = −*RT* ln(*k*_f_/*k*_u_) = 9.5 ± 0.3 kcal mol^−1^. This value was in good agreement with Δ*G*_N2‐I2_ = 10.2 ± 0.9 kcal mol^−1^ calculated from equilibrium chemical denaturation data (Table S1). The sum of kinetic *m*‐values (*m*_f_ + *m*_u_ = 3.3 ± 0.2 kcal mol^−1^ M^−1^) was in excellent agreement with *m*_N2‐I2_ = 3.4 ± 0.2 kcal mol^−1^ M^−1^ obtained from chemical equilibrium denaturation data (Table S1). The good agreement of thermodynamic quantities obtained from kinetics and equilibrium data allowed us to assign with confidence the microscopic rate constants and *m*‐values to the transition N_2_ ↔ I_2_ of the Av‐Flag‐CTD.

### 2.5 N‐terminal helices unfold during formation of the intermediate

To gain insights into structural transitions associated with formation of the intermediate we applied a site‐directed mutagenesis approach. We chose the Lh‐MaSp1‐CTD as a representative homologue. We mutated individual residue side chains to probe their influence on folding or to design an intrinsic fluorescence probe for folding. Deletion mutations were applied probing the energetic contributions of the individual residue side chains to folding and stability. A Trp mutation was applied to generate a local fluorescence probe for folding.

**FIGURE 5:**
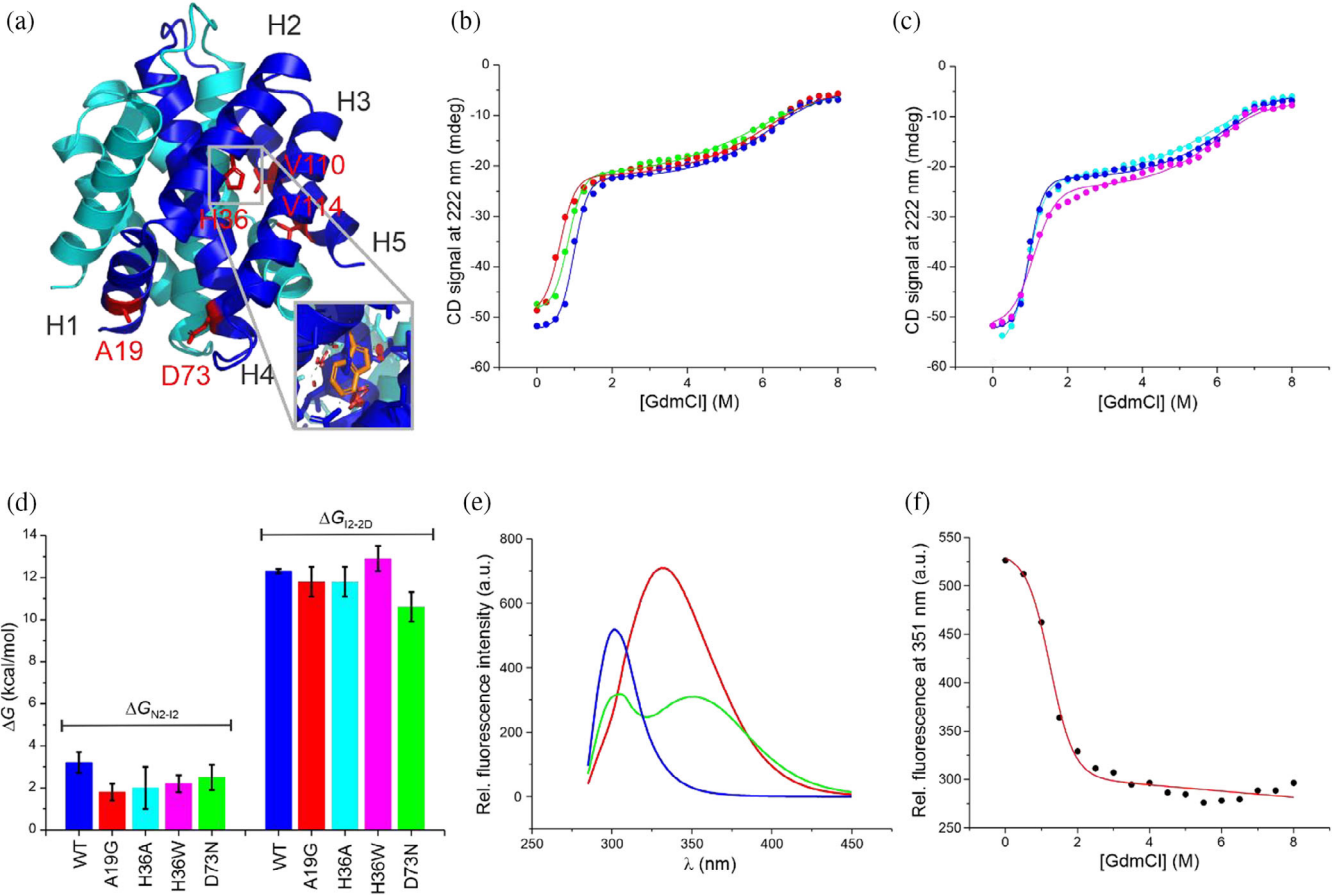
Mutagenesis of Lh‐MaSp1‐CTD. (a) Structural model of the Lh‐MaSp1‐CTD highlighting applied single‐point mutations (red). Monomer subunits are colored in blue and cyan. Helices H1–H5 of one subunit are indicated. Residue side chains subjected to mutagenesis are labeled and shown as red sticks. The expanded view shows the structural model of Trp mutant H36W generated using the mutagenesis tool of PyMOL. The side chain H36W is shown as orange sticks. Steric clashes are shown as red spots. (b) Chemical equilibrium denaturation curves of mutants A19G (red) and D73N (green) are shown together with the wild type (blue) for comparison. (c) Chemical equilibrium denaturation curves of mutants H36A (cyan) and H36W (magenta) are shown together with the wild type (blue) for comparison. Data were recorded at pH 7.0 under reducing conditions using far‐UV CD spectroscopy. Lines are fits to the data using the thermodynamic model for a three‐state transition, N_2_ ↔ I_2_ ↔ 2D. (d) Free energies of unfolding of native and intermediate states of mutants in comparison to the wild‐type protein. (e) UV fluorescence spectra of wild‐type Lh‐MaSp1‐CTD (blue) and mutant H36W (red) measured at pH 7.0, and of mutant H36W measured under reducing conditions in pH 7.0 buffer containing 8 M GdmCl (green). (f) Chemical equilibrium denaturation of mutant H36W measured at pH 7.0 under reducing conditions using Trp fluorescence spectroscopy. The red line is a fit to the data using the thermodynamic model for a two‐state transition.

Alanine (Ala) is known to act as a helix stabilizer (Rohl et al., 1999; Serrano et al., 1992; Spek et al., 1999). The N‐terminus of CTDs is rich in Ala (Figure 1a). The mutation Ala → Gly removes the stabilizing effect and can serve as a probe for helix formation (Lopez‐Llano et al., 2006; Scott et al., 2007; Serrano et al., 1992). We mutated an Ala residue at the centre of helix 1 to Gly (A19G; Figure 5a, the sequence numbering scheme in Figure 1a applies). His at position 36 is at the centre of helix 2 (H36). The side chain of H36 is buried in the hydrophobic core of each monomer. We applied mutation H36A to delete this side chain through mutation to an alanine. Interestingly, an alanine at position 36 is conserved across the investigated MaSp1 and MaSp2 homologues. Only the Lh‐MaSp1‐CTD contains a His at this position (Figure 1a). Visual analysis of the structure using PyMOL (The PyMOL Molecular Graphics System, Version 2.5.0a0 Schrödinger, LLC) indicated that the environment of H36 tolerates substitution to the larger indole side chain of Trp (W) (Figure 5a). This suggested mutation H36W as a fluorescence probe for folding of helix 2 with minimal probe‐induced perturbation. A carboxylic acid side chain at position 73 is located on helix 4, which is part of the dimerization interface, and is conserved across the homologues. This carboxylic acid forms a salt bridge with Arg23 located on helix 1. Lh‐MaSp1‐CTD, instead, contains an Ala at position 23 and a salt bridge thus cannot be formed. To probe a potential effect of the Asp73 (D73) carboxylic acid on folding of Lh‐MaSp1‐CTD we introduced mutation D73N that neutralizes the negative side‐chain charge (Figure 5a). The side chains of Val110 and Val114 are located in buried positions on C‐terminal helix 5. We introduced mutations V110A and V114A individually to probe formation of helix 5. Unfortunately, both mutants failed to express and could thus not be investigated.

Far‐UV CD spectra of mutants A19G, H36A, H36W, and D73N were characteristic of properly folded, all‐helical domains, similar to the wild type (Figure S5). We recorded chemical denaturation data under reducing solution conditions to investigate the influence of mutations on folding and dimerization. Folding was probed using the far‐UV CD spectroscopy. All mutants showed three‐state unfolding transitions, similar to the wild‐type protein (Figure 5b,c). Data were well described by the three‐state model of dimer unfolding, N_2_ ↔ I_2_ ↔ 2D. Thermodynamic parameters obtained from the fits to the data are summarized in Table S5. Changes of free energy are collated in Figure 5d. Δ*G*_N2‐I2_ of mutant A19G was smaller than the value of the wild‐type, but Δ*G*_I2‐2D_ remained within error. Mutants H36A and H36W showed a similar pattern of changes in free energy, that is, reduced values of Δ*G*_N2‐I2_ and Δ*G*_I2‐2D_ that remained within error compared to the wild type. Since residues A19 and H36 are located on helix 1 and on helix 2, respectively, the result indicated that both helices unfold during the first transition N_2_ ↔ I_2_. Mutant D73N showed the opposite effect. Δ*G*_N2‐I2_ of D73N was close to the value of the wild type, but Δ*G*_I2‐2D_ was reduced compared to the wild type and the other mutants. This result indicated that helix 4 unfolded during the second transition.

The Trp fluorescence emission spectra of mutant H36W were characteristic of an indole side chain positioned in a hydrophobic core. Upon chemical denaturation we observed strong quenching of fluorescence emission that was accompanied by a bathochromic shift of the wavelength of maximal fluorescence intensity from originally 333 nm in the native state to 352 nm in the denatured state (Figure 5e). The observed quenching and red‐shift of fluorescence emission is characteristic of a change of the micro‐environment of the Trp side chain from buried to solvent‐exposed. There was an additional fluorescence emission maximum at a wavelength of 300 nm that emerged in the chemically denatured state of mutant H36W. Wild‐type Lh‐MaSp1‐CTD contains four native Tyr residues. Comparison of the fluorescence spectrum of chemically denatured mutant H36W with the spectrum of the wild‐type protein showed that the fluorescence signal originated from native Tyr residues (Figure 5e). We recorded chemical equilibrium denaturation data of mutant H36W using Trp fluorescence as a probe for folding and GdmCl as denaturant. We observed changes of the fluorescence emission intensity that were well described by a thermodynamic model for a mono‐molecular two‐state transition (Figure 5f). The thermodynamic parameters obtained from fits to the fluorescence data of mutant H36W were in very good agreement with the ones of the first unfolding transitions of the same mutant and mutant H36A using far‐UV CD spectroscopy. This showed that fluorescence and far‐UV CD spectroscopy reported on the same conformational change.

## 3 DISCUSSION

The globular C‐terminus of spidroins is a highly conserved (Strickland et al., 2018), pH‐sensitive connectivity module. CTDs have an established role in inducing conformational changes during silk formation facilitated by acid‐induced CTD destabilization, leading to formation of β‐sheet secondary structures (Andersson et al., 2014; Bauer and Scheibel, 2017; Eisoldt et al., 2012; Gauthier et al., 2014; Hagn et al., 2010). Folding studies can shed light on the underlying mechanisms. Previously, urea was applied as a chaotrope in chemical denaturation experiments of CTDs, which led to incomplete unfolding and the observation of residual structure in denatured states (Andersson et al., 2014; Bauer and Scheibel, 2017; Gao et al., 2013; Gauthier et al., 2014; Hagn et al., 2010; Li et al., 2022). In agreement with these results, we found that four of the five investigated CTDs did not fully unfold in solutions containing concentrations of up to 10 M urea (Figure 3). The ability of CTDs to resist such high concentrations of denaturant underscores their remarkable strengths as dimers serving as connectivity modules in silk. Our results show that full unfolding can be achieved by applying the stronger denaturant GdmCl, which allowed us to explore their entire free energy surfaces of folding and dimerization (Figure 2). We found a three‐state mechanism of folding and dimerization via a partially structured, dimeric intermediate that was conserved across five homologues. Intermediates are often transient species in the folding of small protein domains and difficult to detect (Brockwell and Radford, 2007). But here, the intermediates were exclusively populated in denaturation experiments where they were evident as well‐defined intermediate‐state baselines in denaturation curves. Under oxidizing solution conditions, as they exist in web spiders' spinning ducts, partially folded intermediates of CTDs from the Ma glands were the end points of unfolding (Figure 2a–c). Previously, equilibrium and kinetic experiments carried out on the Ea‐MaSp1‐CTD indicated that the first step in folding was the rapid formation of a tight dimer core followed by slow folding of peripheral helices in the second step (Rat et al., 2018). Given that the core of CTD dimers consists of two long, often covalently linked helical stalks, it is reasonable to assume that peripheral helices unfold first during acid‐induced denaturation of the native dimer in a spinning duct. The observed 50%–60% loss of helix in denatured or intermediate states compared to the native state, observed here, adds up with relative sum of residues of the peripheral helices H1–H3 or H1 and H2, and H5, seen in structures (Andersson et al., 2014; Gao et al., 2013; Hagn et al., 2010; Wang et al., 2014). Our mutagenesis experiments indicate that H1 and H2 of native Lh‐MaSp1‐CTD unfold during formation of the intermediate (Figure 5). The fact that constructs containing mutations in the C‐terminal helix H5 failed to express indicated the importance of this C‐terminal helix for structural integrity of the dimer. H5 swaps domains in most of the spidroin CTD dimers characterized so far.

**FIGURE 6:**
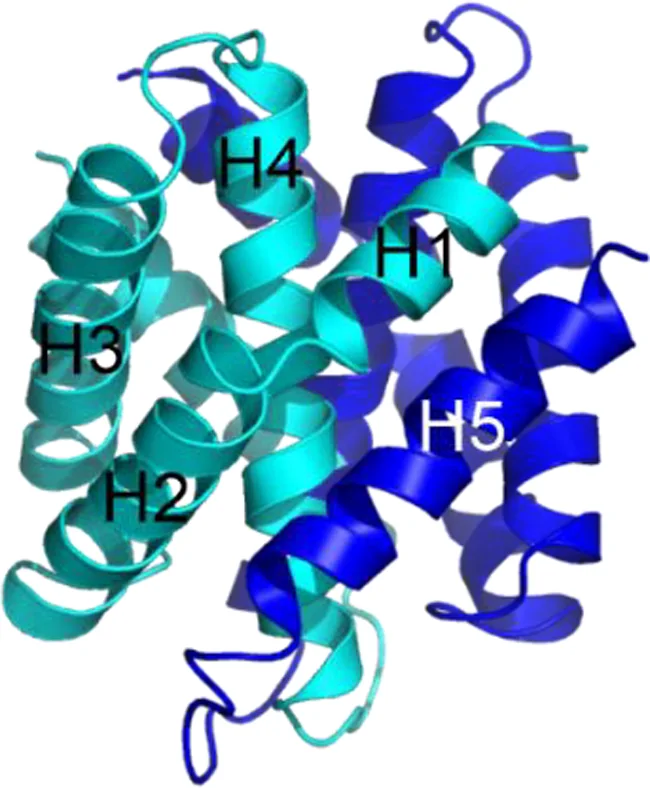
Domain swap of C‐terminal helices creates an interface for docking of N‐terminal helices. Structure of the Lh‐MaSp1‐CTD with monomer subunits colored blue and cyan. Visible helices H1–H4 of one subunit and swapped H5 from the other are indicated. The swapped C‐terminal H5 from the other subunit creates a scaffold for folding and docking of N‐terminal H1 and H2.

Proteins that swap domains upon dimerization contain a pre‐evolved association interface that is shared between monomers (Anonymous, 1994; Bennett et al., 1994). In spidroin CTDs the swap of C‐terminal H5 creates an interface for docking of N‐terminal helices H1 and H2 on the other monomer (Figure 6). H5, H1, and H2 are likely to unfold cooperatively because the helices stabilize each other through quaternary interactions. Transition N_2_ → I_2_ of domain‐swapped spidroin CTDs thus likely involves unfolding of H1, H2, and H5.

**FIGURE 7:**
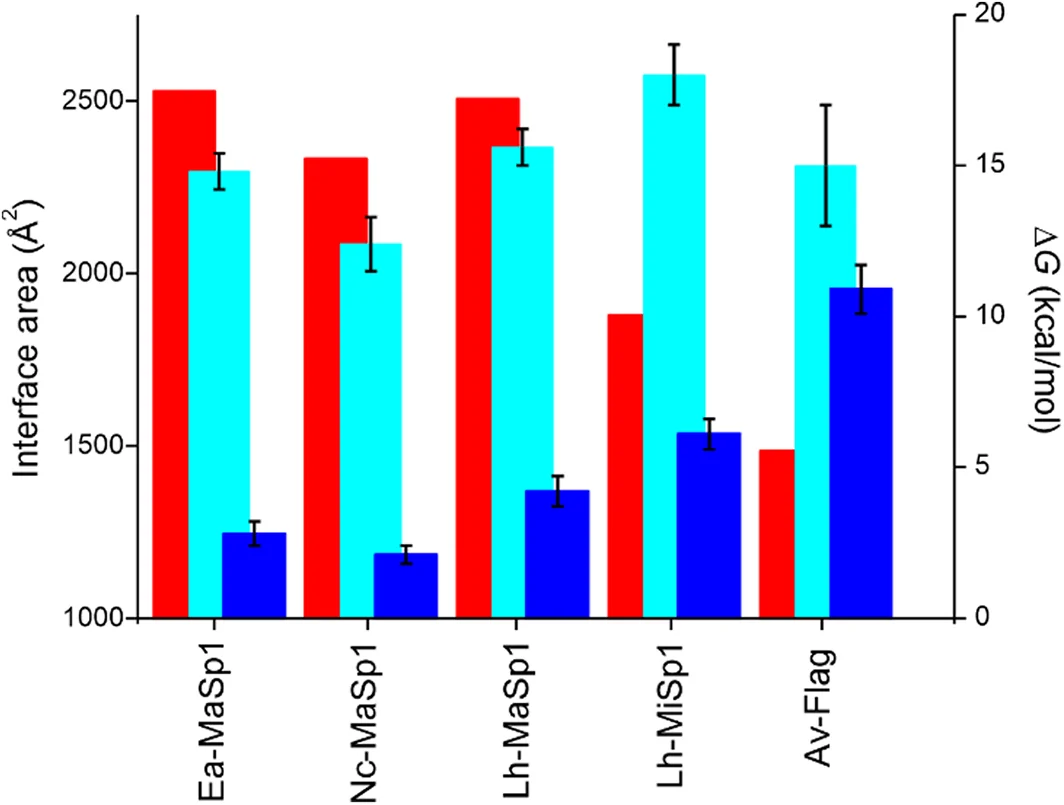
Dimerization interface areas and stabilities of spidroin CTDs. Areas of the dimerization interfaces, calculated using the PISA tool of the Protein Data Bank in Europe (PDBePISA, EMBL‐EBI), are shown in red. The changes of free energy of the transitions N_2_ to I_2_ (Δ*G*_N2‐I2_) and N_2_ to 2D (Δ*G*_N2‐2D_), determined from chemical equilibrium denaturation data recorded in oxidizing and reducing solutions at pH 7.0, are shown in blue and cyan, respectively. Error bars are propagated standard errors from regression analysis.

Structural studies show that domain swap of protein dimers increases the buried surface area of the dimerization interface by ~72% on average (MacKinnon and Wodak, 2015). This substantial increase of buried surface area is thought to increase the stability of the assembly and suggests a biological role of domain swap in protein stabilization (Mackinnon et al., 2013; MacKinnon and Wodak, 2015). We calculated the areas of dimerization interfaces of our investigated CTDs from structures using the PISA tool of the Protein Data Bank in Europe (PDBePISA, EMBL‐EBI) (Figure 7). The domain‐swapped dimerization interfaces of CTDs from the Ma glands were 70%–75% higher than the one of the Av‐Flag‐CTD, in agreement with the reported ~72% expectation value (MacKinnon and Wodak, 2015). But the free energies of unfolding and dissociation (Δ*G*_N2‐2D_) of the five homologues were within error (Figure 7). The stability of the Av‐Flag‐CTD that lacks domain swap was highest among the five homologues, with reference to the formation of the intermediate (Δ*G*_N2‐I2_). This is contrary to the expectation of a stabilizing effect of domain swap on protein stability (Mackinnon et al., 2013; MacKinnon and Wodak, 2015). The finding can be explained by the concept of contact order in protein folding, which suggests that the rate constant of folding is largely determined by the topology of the native structure (Grantcharova et al., 2001). Contact order is defined as the average distance in sequence between residues that form native contacts (Plaxco et al., 1998). The entropic cost of folding increases with increasing sequence separation of interacting residues. Protein structures that exhibit high contact order fold slowly because of this entropic penalty (Grantcharova et al., 2001). In domain swap, native contacts are created between residues of two independent polypeptide chains. This can be viewed as an extreme case of high contact order because a sequence separation is not defined. Our kinetic experiments showed that the rate constant of folding of the Av‐Flag‐CTD was *k*_f_ = 6400 ± 1900 s^−1^, which is four orders of magnitude higher than the value determined for a Ma spidroin CTD of *k*_f_ = 0.78 ± 0.07 s^−1^ (Rat et al., 2018). The rate constant of unfolding of the Av‐Flag‐CTD was *k*_u_ = 0.7 ± 0.1 × 10^−3^ s^−1^, which compares to *k*_u_ = 9 ± 2 × 10^−3^ s^−1^ measured for a Ma CTD (Rat et al., 2018). The higher stability of the Av‐Flag‐CTD compared to the Ma homologue thus originates largely from its higher rate constant of folding (Δ*G* = −*RT* ln(*k*_f_/*k*_u_)). The discrepancy in folding rates is explained by the loss of conformational entropy associated with domain swap. An alternative explanation is that steric hindrance, arising from the volume excluded by the partially folded dimer core formed by helices 3 and 4 in I_2_, slows folding and docking of C‐terminal helix 5 onto the other subunit.

Equilibrium *m*‐values derived from chemical denaturation experiments contain structural information. In chemical denaturation of single‐domain proteins, the equilibrium *m*‐value correlates with the amount of surface exposed to the solvent upon unfolding (Myers et al., 1995). In chemical denaturation of protein dimers, however, the *m*‐value is more difficult to interpret because unfolding involves protein dissociation. Among the five CTDs studied, the Av‐Flag‐CTD was the only homologue that showed a mono‐molecular three‐state transition with well separated native, intermediate, and denatured states populated by a covalent dimer (Figure 2e). Here we could interpret the *m*‐values with confidence. In all other homologues, the covalent dimers resisted unfolding to fully denatured dimers and showed apparent two‐state transitions to structured denatured or intermediate states, except of the Lh‐MiSp1‐CTD that contained no Cys and dissociated to unfolded monomers (Figure 2a–d). For the mono‐molecular three‐state unfolding transition of the Av‐Flag‐CTD, the *m*‐value of the first transition was substantially higher than the *m*‐value of the second transition (Table S1). This indicated that large parts of buried surface area became exposed to solvent in the first unfolding transition. Strikingly, this difference in *m*‐values between the first and second transitions was also evident in three‐state unfolding of all homologues studied under reducing conditions (Table S2). We found that the *m*‐values of the second step were essentially independent on protein concentration (the s.d. of measurements at five different protein concentrations was ±0.1 kcal M^−1^ mol^−1^). The higher s.d. of the mean *m*‐value of the Av‐Flag‐CTD of ±0.3 kcal M^−1^ mol^−1^ can be explained by the fact that the first and second transitions appeared to merge, which led to higher errors of thermodynamic parameters extracted from fits to the data (Table S2, Figure 2e). Our data of five homologues indicated that the largest part of buried surface area of native CTD dimers becomes exposed to solvent in the first unfolding transition. This unfolding transition is relevant for acid‐induced destabilization and subsequent partial unfolding of the CTD along a spider's spinning duct, which is thought to induce structural transitions in spidroins (Andersson et al., 2014; Bauer and Scheibel, 2017; Eisoldt et al., 2012; Gauthier et al., 2014; Hagn et al., 2010). The high *m*‐value associated with transition N_2_ to I_2_ indicates that large parts of structure of CTD dimers are unfolded in I_2_ and ready to refold into, for example, β‐sheet secondary structure.

In conclusion, we found a three‐state mechanism of folding and dimerization that was conserved across homologous spidroin CTDs. In the dimeric intermediate state, large parts of peripheral helices are unfolded. The entropic penalty of domain swap of CTD dimers from Ma and Mi glands, serves to destabilize the assembly for functional purpose. This destabilizing effect contrasts with the common view of a stabilizing effect domain swap exerts on protein dimers through domain swap. Our finding of a destabilizing effect of domain swap of spidroin CTDs is supported by results from structural studies. Soluble spidroins from Ma and Mi glands that contain domain‐swapped CTDs undergo conformational transitions into β‐sheet secondary structures found in silk fibers (Lefevre et al., 2011). The CTD from the Flag gland, instead, lacks domain swap, and the corresponding spidroin undergoes no structural transitions during silk formation (Lefevre et al., 2011). A conformationally more stable CTD lacking domain swap impedes structural transitions.

## 4 METHODS

### 4.1 Protein expression and site‐directed mutagenesis

Synthetic genes of Ea‐MaSp1‐CTD, Nc‐MaSp1‐CTD, Lh‐MaSp1‐CTD, Lh‐MiSp1‐CTD, and Av‐Flag‐CTD were cloned into a modified pRSET A vector (Invitrogen, Thermo Fisher Scientific) using conventional restriction digestion and ligation protocols. Recombinant CTDs were overexpressed in *E. coli* C41 (DE3) bacterial cells as His_6_‐tagged, C‐terminal fusion proteins. To improve yields of synthesis of the constructs Ea‐MaSp1‐CTD, Nc‐MaSp1‐CTD, Lh‐MiSp1‐CTD, and Av‐Flag‐CTD, fusion proteins were inserted between the His_6_‐tag and the CTD, followed by a thrombin recognition sequence for proteolytic removal of the tag and/or fusion protein. For synthesis of the Ea‐MaSp1‐CTD, the lipoyl domain of a pyruvate dehydrogenase multienzyme complex was used as a fusion. For synthesis of the Lh‐MiSp1‐CTD, the apical domain of the molecular chaperone GroEL was used as a fusion (Zahn et al., 1996). For synthesis of the Nc‐MaSp1‐CTD and the Av‐Flag‐CTD, the N‐terminal domain of the molecular chaperone Hsp90 from yeast was used as a fusion (Schulze et al., 2016). Synthesis of the Lh‐MaSp1‐CTD did not require the application of a fusion protein. Single‐point mutants of the Lh‐MaSp1‐CTD (A19G, H36A, H36W, and D73N) and of the Av‐Flag‐CTD (mutant C105S) were generated using the QuikChange mutagenesis protocol (Stratagene). CTDs and mutants thereof were isolated from clarified cell lysate by affinity chromatography using Ni‐Sepharose 6 Fast‐Flow resin (GE Healthcare). Proteolytic cleavage of the His_6_‐tag‐fusion was carried out over‐night at room temperature using thrombin from bovine plasma (Sigma‐Aldrich) and during dialysis against aqueous buffered solutions for the next chromatography step applied to remove fusion proteins. Constructs Ea‐MaSp1‐CTD, Nc‐MaSp1‐CTD, and Lh‐MiSp1‐CTD were dialyzed against 50 mM phosphate pH 8.0 containing 30 mM imidazole, followed by a second Ni‐Sepharose affinity chromatography. The Av‐Flag‐CTD was dialyzed against 20 mM Tris–HCl, pH 8.0, followed by ion exchange chromatography (Q Sepharose Fast‐Flow resin, Cytiva). Proteins were purified to homogeneity using size exclusion chromatography (SEC) on a Superdex 75 column (Cytiva) equilibrated with 200 mM ammonium bicarbonate. Purity of the isolated CTDs was confirmed using SDS‐PAGE. Pooled protein fractions from SEC were lyophilized and stored at −20°C.

### 4.2 Far‐UV CD spectroscopy and chemical denaturation experiments

Far‐UV CD spectroscopy was carried out using a J‐815 spectropolarimeter (Jasco) and a 1 mm path‐length cuvette (Hellma). Sample temperature was set to 298 K throughout all experiments using a Peltier thermocouple. Spectra were recorded at protein concentrations of typically 15 μM in 50 mM phosphate, pH 7.0, with the solution ionic strength adjusted to 200 mM using potassium chloride. Denaturation of CTDs was probed at 222 nm, the wavelength of the maximal signal amplitude of α‐helix secondary structure. Chemical denaturation of 20 μM protein samples was performed by manual titration between 0 and 10 M urea in 50 mM phosphate, pH 7.0, with the solution ionic strength adjusted to 200 mM using potassium chloride, and in 20 mM 2‐(*N*‐morpholino)ethanesulfonic acid (MES), pH 5.7, with the solution ionic strength adjusted to 200 mM using potassium chloride. Reducing solution conditions were generated by adding 1 mM DTE to buffered solutions. Denaturation experiments using GdmCl as denaturant were performed by manual titration between 0 and 8 M GdmCl in 50 mM MOPS, pH 7.0. Concentration‐dependent denaturation experiments were carried out by manual titration of samples containing between 5 and 40 μM protein.

### 4.3 Steady‐state fluorescence spectroscopy

Steady‐state UV fluorescence spectroscopy was carried out on a FP‐8350 spectrofluorometer (Jasco) using a 10‐mm pathlength fluorescence cuvette (Hellma). Fluorescence of CTD samples was excited at 277 nm. The temperature was set to 298 K throughout all measurements using a Peltier thermocouple. Sample concentration was 10 μM protein. Chemical denaturation experiments were carried out by manual titration between 0 and 8 M GdmCl in 50 mM MOPS, pH 7.0, containing 1 mM dithiothreitol (DTT).

### 4.4 Stopped‐flow spectroscopy

Kinetics of folding were measured by following the time course of the far‐UV CD signal at 222 nm in rapid‐mixing experiments. A SFM‐2000 stopped‐flow machine (BioLogic Instruments) was coupled to a J‐815 spectropolarimeter (Jasco). Sample temperature was controlled using a circulating water bath set to 298 K throughout all experiments. Measurement of kinetics of folding/unfolding (transition N_2_ ↔ I_2_) of the Av‐Flag‐CTD was carried out under oxidizing conditions in 50 mM MOPS, pH 7.0. Kinetics of unfolding were measured by mixing 0.5 mM native Av‐Flag‐CTD in 50 mM MOPS, pH 7.0, containing various concentrations of GdmCl. Kinetics of folding were measured by mixing 0.5 mM chemically denatured Av‐Flag‐CTD in 50 mM MOPS, pH 7.0, containing various concentrations of GdmCl. The applied mixing ratio was 1:10. Typically 10 shots were averaged to obtain transients of sufficient signal‐to‐noise for kinetic analysis.

### 4.5 Data analysis

Equilibrium chemical denaturation data were fitted using thermodynamic models for two‐state or three‐state mechanisms of folding and dimerization, which are outlined below.

#### 4.5.1 Reaction scheme N_2_ ↔ I_2_

Transitions between a native dimer N_2_ and a dimeric intermediate I_2_ were fitted using equations based on the classical model for a two‐state equilibrium between native and denatured conformational states. The spectroscopic signal *S* can be expressed as a function of denaturant concentration (Santoro and Bolen, 1988):

(1) $$S\left(\mathrm{Den}\right)=\frac{\alpha_{N}+\beta_{N}∙\left[\mathrm{Den}\right]+\left(\alpha_{D}+\beta_{D}∙\left[\mathrm{Den}\right]\right)∙\exp\left(-\frac{∆G_{D-N}}{\mathrm{RT}}\right)}{1+\exp\left(-\frac{∆G_{D-N}}{\mathrm{RT}}\right)},$$

where *α*_N_, *β*_N_, *α*_D_, and *β*_D_ are the signals of the native (N_2_) and the denatured (I_2_) state, respectively, that change linearly with denaturant concentration ([Den]); *R* is the gas constant and *T* is the temperature. The free energy of folding changes linearly with denaturant concentration (Tanford, 1968):

(2) $${\Delta G}_{D-N}\left(\left[\mathrm{Den}\right]\right)=∆G_{D-N}-m_{D-N}\left[\mathrm{Den}\right],$$

where Δ*G*_D‐N_ is the free energy of folding in aqueous solution without denaturant and *m*_D‐N_ is the equilibrium *m*‐value.

#### 4.5.2 Reaction scheme N_2_ ↔ I_2_ ↔ 2D

Transitions between a native dimer N_2_, a dimeric intermediate I_2_, and denatured monomers D were described by a thermodynamic three‐state model of folding and dimerization involving a dimeric intermediate (Hobart et al., 2002). The spectroscopic signal is expressed as the sum of the signals of N_2_ (*S*_N2_), I_2_ (*S*_I2_), and D (*S*_D_):

(3) $$S=S_{N}∙\left(\frac{2P_{t}∙F_{D}^{2}}{K_{1}∙K_{2}}\right)+S_{I}∙\left(\frac{2P_{t}∙F_{D}^{2}}{K_{2}}\right)+S_{D}∙\left(F_{D}\right),$$

where *P*_t_ is the total protein concentration in terms of monomer and *F*_D_ is the fraction of denatured monomer.

The fraction of denatured monomer, *F*_D_, is expressed as (Hobart et al., 2002):

(4) $$F_{D}=\frac{-K_{1}∙K_{2}\sqrt{{\left(K_{1}∙K_{2}\right)}^{2}+8\left(1+K_{1}\right)∙\left(K_{1}∙K_{2}\right)∙P_{t}}}{4P_{t}∙\left(1+K_{1}\right)}.$$

The equilibrium constants for the first transition (N_2_ ↔ I_2_), *K*_1_, and for the second transition (I_2_ ↔ 2D), *K*_2_, are defined as follows:

(5) $$\begin{array}{l} K_{1}=\exp\left(\frac{m_{N2-I2}∙\left(\left[\mathrm{Den}\right]-{\left[\mathrm{Den}\right]}_{50\%}^{N2-I2}\right)}{\mathrm{RT}}\right);\\ K_{2}=\exp\left(\frac{-∆G_{I2-2D}+m_{I2-2D}∙\left[\mathrm{Den}\right]}{\mathrm{RT}}\right), \end{array}$$

where *m*_N2‐I2_ is the equilibrium *m*‐value of the first transition, ${\left[\mathrm{Den}\right]}_{50\%}^{N2-I2}$ is the mid‐point concentration of denaturant of the first transitions, Δ*G*_I2‐2D_ is the free energy of the second transition, and *m*_I2‐2D_ is the equilibrium *m*‐value of the second transition.

Δ*G*_N2‐I2_ was calculated following the free energy relationship (Tanford, 1968):

(6) $$∆G_{N2-I2}=m_{N2-I2}∙{\left[\mathrm{Den}\right]}_{50\%}^{N2-I2}.$$

#### 4.5.3 Reaction scheme N_2_ ↔ I_2_ ↔ D_2_

Three‐state unfolding of the Av‐Flag‐CTD measured under oxidizing conditions, retaining a covalent dimer, was described by a monomolecular three‐state model (Walters et al., 2009). In this model, the spectroscopic signal *S* is defined by the sum of the signal N_2_ (*S*_N2_), I_2_ (*S*_I2_), and D_2_ (*S*_D2_):

(7) $$S=\frac{S_{N2}+S_{I2}*K_{1}+S_{D2}*K_{1}*K_{2}}{\left(1+K_{1}+K_{1}*K_{2}\right)}.$$

*S*_N2_, *S*_I2_, and *S*_D2_ were described as linearly sloping baselines *α*_N2_ + *β*_N2_[Den], *α*_I2_ + *β*_I2_[Den], and *α*_D2_ + *β*_D2_[Den], like in Equation (1). The equilibrium constants of the first transition (N_2_ ↔ I_2_), *K*_1_, and for the second transition (I_2_ ↔ D_2_), *K*_2_, were expressed by the corresponding free energies Δ*G*_N2‐I2_ and Δ*G*_I2‐D2_, respectively, using the linear free energy relationship (Tanford, 1968), as described above.

#### 4.5.4 Reaction scheme N_2_ ↔ 2D

A two‐state transition between a non‐covalent, native dimer (N_2_) and denatured monomers (D) was described by a thermodynamic model where the fraction of denatured monomer, *F*_D_, is expressed as (Hobart et al., 2002):

(8) $$F_{D}=\frac{\sqrt{{K_{N2-D}}^{2}+8K_{N2-D}∙P_{t}}-K_{N2-D}}{4P_{t}}.$$

The equilibrium constant *K*_N2‐D_ is defined as *K*_N2‐D_ = [D]^2^/[N_2_] and *P*_t_ is the total protein concentration in terms of monomer (Hobart et al., 2002). *K*_N2‐D_ was expressed by the corresponding free energy Δ*G*_N2‐D_ involving the linear free energy relationship. The spectroscopic signal *S* was defined by the sum of the signals of N_2_ and D weighted by their corresponding fractions (Hobart et al., 2002):

(9) $$S=S_{N2}∙\left(1-F_{D}\right)+S_{D}∙\left(F_{D}\right).$$

*S*_N2_ and *S*_D_ were modeled as linearly sloping baselines *α*_N2_ + *β*_N2_[Den] and *α*_D_ + *β*_D_[Den].

#### 4.5.5 Kinetics of folding

Kinetic transients measured using stopped‐flow far‐UV CD spectroscopy were fitted to mono‐exponential or bi‐exponential decay functions:

(10) $$\begin{array}{l} S\left(t\right)=a∙\exp\left(-k_{\mathrm{obs}}∙t\right)+b;\\ S\left(t\right)=a_{1}∙\exp\left(-k_{\mathrm{obs}1}∙t\right)+a_{2}∙\exp\left(-k_{\mathrm{obs}2}∙t\right)+b, \end{array}$$

where *S*(*t*) is the spectroscopic signal recorded as function of time, *a* and *k*_obs_ are the amplitude and the observed rate constant of the obtained decay, and *b* is the signal of the baseline. *k*_obs_ contains the sum of microscopic rate constants of folding and unfolding (*k*_f_ + *k*_u_). *k*_obs_ was analyzed as a function of denaturant concentration by fitting a chevron model for a barrier‐limited two‐state transition following the linear‐free‐energy relationship (Jackson and Fersht, 1991):

(11) $$\log k_{\mathrm{obs}}\text{([GdmCl])}=\begin{array}{l} \log[k_{f}\exp\left(-m_{f}\frac{\text{[GdmCl]}}{\mathrm{RT}}\right)\\ +\;k_{u}\exp\left(m_{u}\frac{\text{[GdmCl]}}{\mathrm{RT}}\right)], \end{array}$$

where *m*_f_ and *m*_u_ are the microscopic *m*‐values for folding and unfolding, and *k*_f_ and *k*_u_ are the microscopic rate constants of folding and unfolding, respectively, in the absence of denaturant. Errors of reported kinetic parameters are s.e. from regression analysis and propagated s.e.

## AUTHOR CONTRIBUTIONS

Charlotte Rat designed experiments, performed experiments, analyzed data, and wrote the paper; Cedric Heindl performed experiments and analyzed data; Hannes Neuweiler conceptually designed the research, designed experiments, analyzed data, and wrote the paper.

## CONFLICT OF INTEREST STATEMENT

The authors declare no conflicts of interest.

## Supporting information


**Data S1.** Supporting Information.

## Data Availability

The data that support findings of this study are available from the corresponding author upon reasonable request.
