# Supplementary material for: Domain swap facilitates structural transitions of spider silk protein C‐terminal domains
Source: Protein Sci. 2023 Nov 1;32(11):e4783. doi: 10.1002/pro.4783 (PMC10578117; doi:10.1002/pro.4783)
Supplement: Supplementary file 1 — Data S1. Supporting Information. [file PRO-32-e4783-s001.pdf]

Supporting Information

Domain swap facilitates structural transitions of spider silk protein C-terminal domains

Charlotte Rat, Cedric Heindl & Hannes Neuweiler*

Department of Biotechnology and Biophysics, Julius-Maximilians-University Würzburg, Am Hubland, 97074 Würzburg, Germany

*Corresponding author: hannes.neuweiler@uni-wuerzburg.de

Supporting Tables

Supporting Table 1: Thermodynamic parameters of folding measured at pH 7.0 under oxidising conditions using GdmCl as denaturant.

	$[GdmCl]^{50\%}_{N2-I2}$ (M)	m_{N2-I2} (kcal mol^{-1} M^{-1})	ΔG_{N2-I2} (kcal mol^{-1})	$[GdmCl]^{50\%}_{I2-D2}$ (M)	m_{I2-D2} (kcal mol^{-1} M^{-1})	ΔG_{I2-D2} (kcal mol^{-1})
Ea-MaSp1-CTD	0.7±0.1	3.1±0.2	2.2±0.5	n.d.	n.d.	n.d.
Nc-MaSp1-CTD	0.4±0.1	4.4±0.3	1.8±0.6	n.d.	n.d.	n.d.
Lh-MaSp1-CTD	1.4±0.1	3.0±0.2	4.2±0.6	n.d.	n.d.	n.d.
Lh-MiSp1-CTD	0.8±0.1	5.3±0.1	4.2±0.6	n.d.*	n.d.*	n.d.*
Av-Flag-CTD	3.0±0.1	3.4±0.2	10.2±0.9	5.2±0.1	1.8±0.2	9±1

Errors are s.e. from regression analyses; n.d.: not determined; *: since Lh-MiSp1-CTD was a non-covalent dimer, thermodynamics of the bi-molecular second transition ($I_2 \leftrightarrow 2D$) were determined from concentration-dependent denaturation experiments (Supplementary Table 2).

Supporting Table 2: Thermodynamic parameters of folding and dimerization measured at pH 7.0 under reducing conditions using GdmCl as denaturant.

	$[GdmCl]^{50\%}_{N2-I2}$ (M)	m_{N2-I2} (kcal mol^{-1} M^{-1})	ΔG_{N2-I2} (kcal mol^{-1})	ΔG_{I2-2D} (kcal mol^{-1})	ΔG_{N2-2D} (kcal mol^{-1})
Ea-MaSp1-CTD	0.8±0.1	3.4±0.2	2.6±0.4	12.2±0.2	14.8±0.6
Nc-MaSp1-CTD	0.4±0.1	4.4±0.2	1.8±0.6	10.5±0.3	12.4±0.9
Lh-MaSp1-CTD	1.1±0.1	3.1±0.2	3.2±0.5	12.3±0.1	15.6±0.6
Lh-MiSp1-CTD*	0.8±0.1	5.3±0.4	4.2±0.6	13.7±0.2	18±1
Av-Flag-CTD	2.3±0.1	2.8±0.3	6.4±0.6	9±1	15±2

*Lh-MiSp1-CTD was measured under oxidising conditions because it lacks native Cys residues. Values are the mean of five measurements carried out at varying protein concentrations (see Methods) ±s.d.

Supporting Table 3: Thermodynamic parameters of folding measured under oxidising conditions at pH 7.0 and pH 5.7, and under reducing conditions at pH 7.0, using urea as denaturant.

Conditions		Ea-MaSp1-CTD	Nc-MaSp1-CTD	Lh-MaSp1-CTD	Lh-MiSp1-CTD	Av-Flag-CTD
Oxidising pH 7.0	$[\mathrm{GdmCl}]^{50\%}_{\mathrm{N2-I2}}$ (M)	2.5±0.1	1.5±0.1	3.8±0.1	2.9±0.1	6.8±0.2
	$m_{\mathrm{N2\text{-}I2}}$ (kcal mol^{-1} M^{-1})	1.1±0.1	1.4±0.1	1.1±0.1	2.1±0.1	1.6±0.1
	$\Delta G_{\mathrm{N2\text{-}I2}}$ (kcal mol^{-1})	2.8±0.4	2.1±0.3	4.2±0.5	6.1±0.5	10.9±0.8
Oxidising pH 5.7	$[\mathrm{GdmCl}]^{50\%}_{\mathrm{N2-I2}}$ (M)	1.9±0.1	0.9±0.1	2.4±0.1	1.4±0.1	5.3±0.1
	$m_{\mathrm{N2\text{-}I2}}$ (kcal mol^{-1} M^{-1})	1.0±0.1	1.5±0.1	1.2±0.1	2.5±0.2	1.5±0.1
	$\Delta G_{\mathrm{N2\text{-}I2}}$ (kcal mol^{-1})	1.9±0.3	1.4±0.2	2.9±0.4	3.5±0.4	8.0±0.7
Reducing pH 7.0	$[\mathrm{GdmCl}]^{50\%}_{\mathrm{N2-I2}}$ (M)	2.7±0.1	1.2±0.1	2.9±0.3	n.d.	4.7±0.1
	$m_{\mathrm{N2\text{-}I2}}$ (kcal mol^{-1} M^{-1})	1.2±0.1	1.5±0.1	1.5±0.1	n.d.	1.5±0.1
	$\Delta G_{\mathrm{N2\text{-}I2}}$ (kcal mol^{-1})	3.2±0.4	1.8±0.3	4.4±0.4	n.d.	7.1±0.6

Values are from fits to the data ±s.e.; n.d.: not determined.

Supporting Table 4: Thermodynamic parameters of folding and dimerization of mutants of Lh-MaSp1-CTD measured at pH 7.0 under reducing conditions using GdmCl as denaturant.

	$[\mathrm{GdmCl}]^{50\%}_{\mathrm{N2-I2}}$ (M)	$m_{\mathrm{N2\text{-}I2}}$ (kcal mol^{-1} M^{-1})	$\Delta G_{\mathrm{N2\text{-}I2}}$ (kcal mol^{-1})	$\Delta G_{\mathrm{I2\text{-}2D}}$ (kcal mol^{-1})	$\Delta G_{\mathrm{N2\text{-}2D}}$ (kcal mol^{-1})
A19G	0.6±0.1	2.9±0.2	1.8±0.4	11.8±0.7	14±1
D73N	0.8±0.1	3.1±0.3	2.5±0.6	10.6±0.7	13±1
H36W*	1.2±0.1	1.8±0.3	2.2±0.5	n.a.	n.a.

Values are from fits to chemical denaturation data recorded using CD spectroscopy ±s.e.; *values are from Trp fluorescence measurements; n.a.: not applicable.

Supporting Figures

Supporting Figure 1

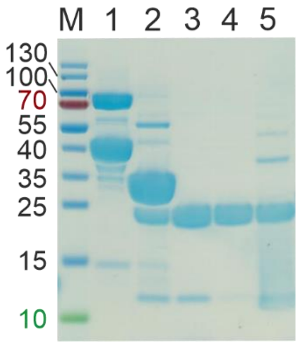

Supporting Figure 1: **SDS-PAGE analysis of synthesis of Av-Flag-CTD**. M: molecular weight markers (kDa). Lanes 1-4: Synthesis of Av-Flag-CTD mutant C105S. Lane 1: eluate of Ni-NTA chromatography; lane 2: proteolytic thrombin digestion of Ni-NTA eluate; lane 3: eluate from ion exchange chromatography after thrombin digestion; lane 4: eluate from size exclusion chromatography after ion exchange chromatography. Lane 5: Correspondingly synthesized wild-type Av-Flag-CTD after four days in solution at room temperature. The sample contained monomeric and oligomeric protein besides a dimer.

Supporting Figure 2

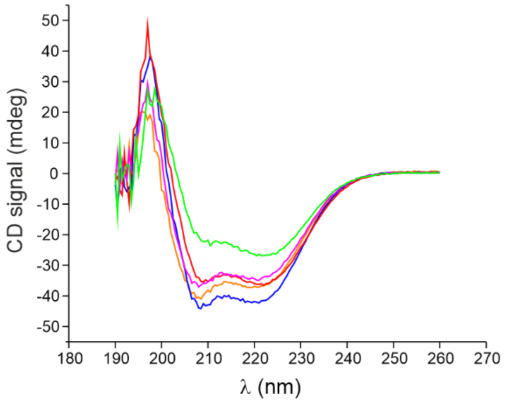

Supporting Figure 2: **Far-UV CD spectra of CTDs**. Far-UV CD spectra of 15 µM samples of Ea-MaSp1-CTD (orange), Nc-MaSp1-CTD (blue), Lh-MaSp1-CTD (red), Lh-MiSp1-CTD (magenta) and Av-Flag-CTD (green) recorded at 25 °C in 50 mM phosphate, pH 7.0, with the solution ionic strength adjusted to 200 mM using potassium chloride.

Supporting Figure 3

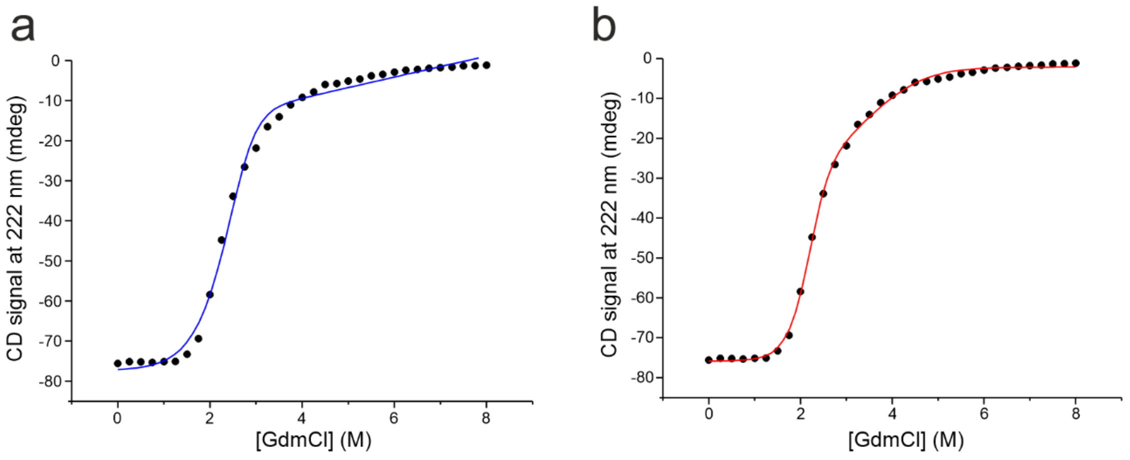

Supporting Figure 3: **Application of two-state versus three-state models of unfolding to denaturation data of the Av-Flag-CTD**. Chemical denaturation data of 40 µM Av-Flag-CTD recorded at pH 7.0 under reducing solution conditions (black circles). (**a**) Fit to the data using a two-state model of dimer denaturation, $N_2 \leftrightarrow 2D$ (blue line). (**b**) Fit to the data using a three-state model of dimer denaturation, $N_2 \leftrightarrow I_2 \leftrightarrow 2D$ (red line).

Supporting Figure 4

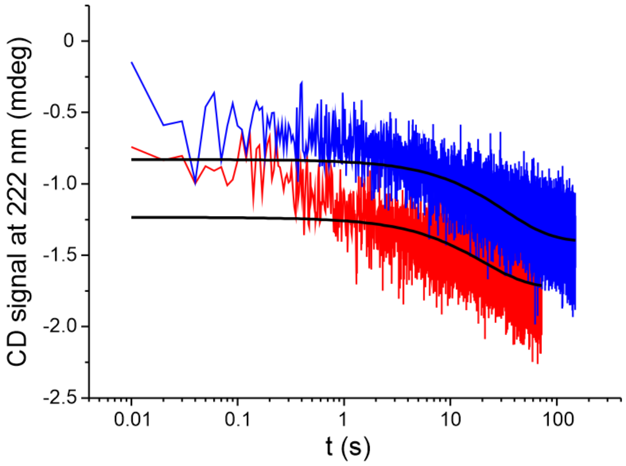

Supporting Figure 4: **Mono-exponential fits to kinetic transients of folding of the Av-Flag-CTD**. Kinetic transients of folding measured in 2.4 M GdmCl and in 2.9 M GdmCl are shown in red and blue. Measurements were performed in pH 7.0 buffered solutions under oxidising conditions. Black lines are mono-exponential fits to the data.

Supporting Figure 5

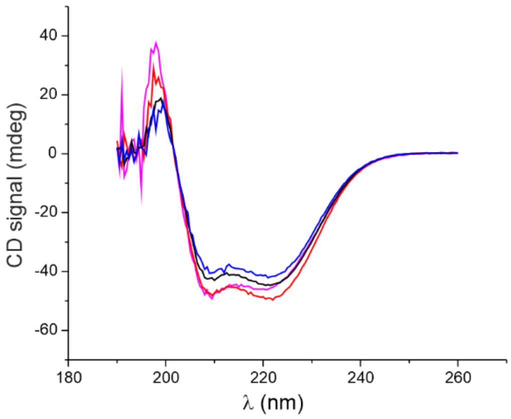

Supporting Figure 5: **Far-UV-CD spectra of single-point mutants of Lh-MaSp1-CTD**. Spectra of 20 µM samples of mutants A19G (blue), H36W (magenta) and D73N (red) recorded at 25 °C in 50 mM phosphate, pH 7.0, with the solution ionic strength adjusted to 200 mM using potassium chloride. The spectrum recorded from the wild-type protein is shown for comparison (black).
